# A new species of the genus *Karnyothrips* (Thysanoptera, Phlaeothripidae) from China

**DOI:** 10.3897/zookeys.346.6216

**Published:** 2013-11-01

**Authors:** Jun Wang, Majid Mirab-balou, Xiao-li Tong

**Affiliations:** 1 College of Plant Science, Jilin University, Changchun 130062, China; 2 Department of Plant Protection, College of Agriculture, Ilam University, Ilam, Iran; 3 Department of Entomology, South China Agricultural University, Guangzhou 510642, China; 4 Northeast Institute of Geography and Agroecology, Chinese Academy of Sciences, Changchun 130102, China

**Keywords:** Litter thrips, Phlaeothripidae, *Karnyothrips*, new species, China

## Abstract

*Karnyothrips cyathomorphus* **sp. n.** (Phlaeothripidae: Phlaeothripinae) is described as a new apterous species in the genus *Karnyothrips* Watson 1923, and it represents the fourth species of the genus to be recorded from China. A key to the Chinese species is given.

## Introduction

The genus *Karnyothrips*, belonging to the *Haplothrips* lineage in Phlaeothripinae, was established by Watson for the species *Karynia weigeli*, a synonym of *Karnyothrips flavipes* (Jones). Currently 47 species have been described in the genus (ThripsWiki 2013), of which three species are recorded from China (Mirab-balou et al. 2011). *Karnyothrips flavipes* is widely distributed in the world, *Karnyothrips melaleucus* (Bagnall) is distributed in tropics and subtropics, and *Karnyothrips robustus* Okajima has been found only in Japan and Taiwan of China.

The *Karnyothrips* species usually live on live plant leaves, branches and dead forest litter, where they feed on micro-invertebrates or fungi. Recently, a distinct new species of *Karnyothrips* has been found while studying the litter thrips fauna in subtropical and tropical China. Specimens were mounted into Canada balsam and deposited in the Insect Collection, Department of Entomology, South China Agricultural University (SCAU).

The diagnosis of the genus includes the following features (Watson 1922; Mound and Marullo 1996; Okajima 2006): head longer than broad, rarely produced in front of eyes; antennae eight segmented, segments VII and VIII broadly joined, segment III variable in shape and usually shorter than segment IV; maxillary stylets especially long and extended into base of postocular setae, maxillary bridge present; pronotum anteromarginal setae often reduced; basantra and ferna usually developed; fore wing if developed, constricted medially, duplicated cilia present; anal setae especially long, much longer than tube.

### Key to *Karnyothrips* species in China

**Table:** 

1	Tergite IX S_1_ setae longer than tube length, apex pointed [Taiwan, Yunnan, Guizhou, Fujian, Guangdong, Guangxi, Hainan]	*Karnyothrips melaleucus* (Bagnall)
–	Tergite IX S_1_ setae shorter than tube length, apex expanded	2
2	Antennal segment III with a ring-like swelling at base [Guangdong]	*Karnyothrips cyathomorphus* sp. n.
–	Antennal segment III without a ring-like swelling at base	3
3	Antennal segment III with 2 sense cones [Sichuan, Hunan, Guangdong, Guangxi, Hainan, Fujian]	*Karnyothrips flavipes* (Jones)
–	Antennal segment III with 3 sense cones [Taiwan]	*Karnyothrips robustus* Okajima

## Taxonomy

### Karnyothrips cyathomorphus sp. n.

http://zoobank.org/1ED76766-7E7B-4CEC-8045-3037DD74D9A9

http://species-id.net/wiki/Karnyothrips_cyathomorphus

Figs 1−8

#### Specimens examined.

**Holotype:** female. **CHINA**: Guangdong Province, Guangzhou, Botanical Garden of South China Agricultural University (23°09'25"N, 113°21'18"E), from leaf-litter, 15.xii.2004, leg. Jun Wang. **Paratypes:** 2 females and 3 males, same data as holotype; 1 female and 4 males, same locality, habitat and collector, 20.xi.2004; 1 male; Longdong (23°14'07"N, 113°24'05"E), from leaf-litter of *Acacia mangium* plantations, 5.xii.2007, leg. Jun Wang.

#### Description.

**Figures 1−8.**
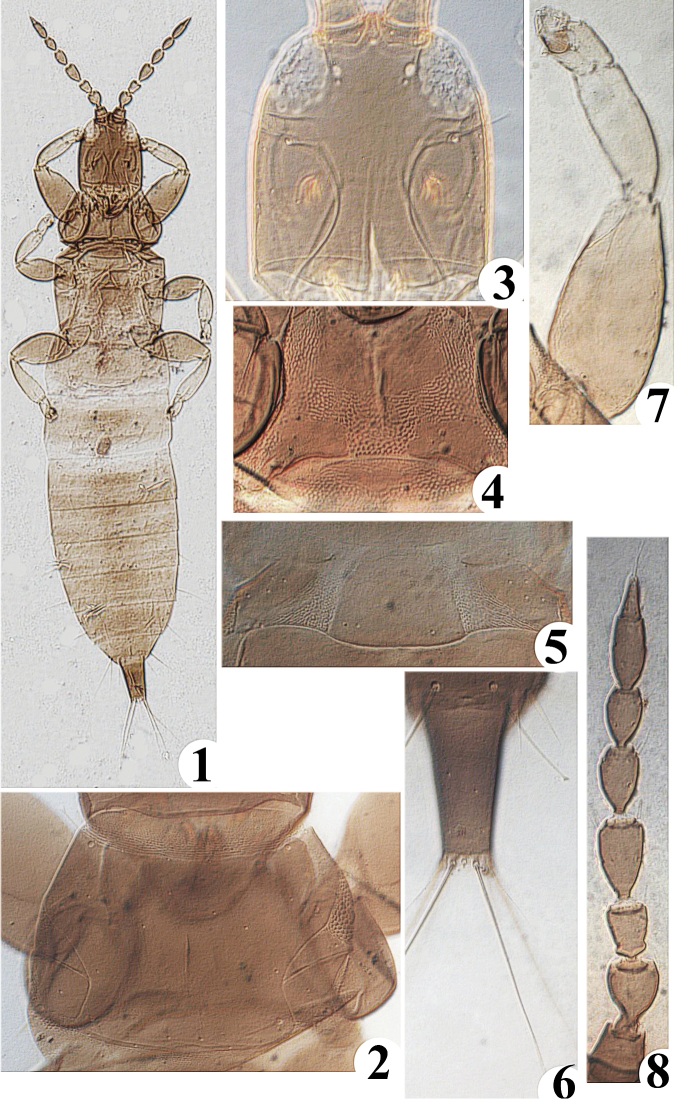
*Karnyothrips cyathomorphus* sp. n. **1** slide mounted apterous adult (dorsal view), female **2** pronotum, female **3** head, female **4** basantra and ferna, female **5** pelta, female **6** abdominal tergum IX and tube, male **7** fore leg, male **8** antenna, female.

Female apterous (Figs 1). Body color brown; head dorsum, pronotum, mesonotum anterior margin, and tube brown, abdominal each tergite anterior margin in middle a little brown the others yellow. Antenna brown, but segment III somewhat paler. All femur brown, tibia and tarsus yellow, fore tibia outer margin a lot darker.

*Head*: dorsum (Figs 3) about 1.4 times as long as broad and little projecting in front of eyes, dorsal surface smooth in the middle and between the eyes, only transverse line sculpture at basal and two sides area; postocular setae distinctly shorter than eye length, apex expanded; cheeks margins subparallel, gradually broader and not constricted behind eyes; eyes round, about 0.3 times as long as head length; ocelli small and posterior far away separated; antennae eight-segmented (Fig. 8) about 1.7 times as long as head length, smooth on surface of each segment; segment III short and constricted at base with a pronounced ring-like swelling; segments III−IV with 2 and 4 sense cones respectively; segment VII elongate and shorter than segment IV, segments VII and VIII broadly joined; maxillary stylets long and extended into base of postocular setae, gradually nearer in the middle, maxillary bridge present.

*Thorax*: Pronotum (Fig. 2) at middle 0.8 time as long as head length, surface smooth, with median longitudinal line; notopleural sutures complete; anteromarginal setae reduced; anteroangulars, midlaterals, posteroangulars and epimeral setae developed, apex expanded; basantra and ferna developed (Fig. 4); all femur enlarged, fore tarsus without tooth.

*Abdomen*: Pelta (Fig. 5) semicircle-shaped and sculptured anteriorly, smooth medially and posteriorly, without lateral lobes, a pair of campaniform sensilla present; tergites II−VII each with two pairs of developed wing-retaining setae; S_1_ setae on tergite IX (Fig. 6) shorter than tube length, apex expanded, S_2_ setae longer than tube, apex sharp; tube almost 0.6 times as long as head length, 1.6 times of tube width; anal setae long and about 1.5 times as long as tube length.

#### Measurements, holotype female in micrometers.

Total body length 1275; head L/W (153/140); eyes length 48, diameter of ocelli 5; distance of posterior ocelli 34; pronotum median length 119, width 231; tube length 89, tube maximum width 56, apex width 35. Antennal segments I–VIII length (width) as follows: 25(29); 33(26); 26(21); 40(25); 35(21); 31(19); 38(15); 23(11). Postocular setae 34; antennal terminal setae 20; pronotum anteroangular setae 31, midlateral setae 31, posteroangular setae 31, epimeral setae 39; tergum IX S_1_ setae 64, S_2_ setae 125.

*Apterous male*: Color and structure similar to apterous female. Major setae on head, pronotum and abdomen capitate except that setae S_2_ on tergite IX are short and pointed, and S_3_ finely acute (Fig. 6). Fore femur well developed and fore tarsal with small tooth (Fig. 7). Abdominal sternites without any glandular area.

#### Measurements, paratype male in micrometers.

Total body length 1263; head L/W (144/138); eyes length 48, diameter of ocelli 4; distance of posterior oceelli 34; pronotum median length 110, width 213; tube length 85, tube maximum width 56, apex width 38. Antennal segments I–VIII length (width) as follows: 19(28); 29(25); 26(21); 39(25); 34(21); 29(19); 38(14); 24(11). Postocular setae 29; antennal terminal setae 16; pronotum anteroangular setae 28, midlateral setae 28, posteroangular setae 28, epimeral setae 35; tergum IX S_1_ setae 60, S_2_ setae 26.

#### Etymology.

The specific epithet is a combination of Latin words cyatho and morphus, referring to the shape of antennal segment III.

#### Distribution.

China (Guangdong).

#### Remarks.

*Karnyothrips cyathomorphus* sp. n. resembles *Karnyothrips inflatus* Okajima in having a sub-basal ring-like swelling on antennal segment III, but it can be distinguished from the latter by the following features: (1) no wing; (2) antennal segment IV with 4 sense cones, antennal segment VII shorter than segment IV; (3) pelta semicircle-shaped and smooth medially and posteriorly. The new species is also similar in appearance to *Priesneria kellyana* Bagnall which is apterous and antennal segment III with a sub-basal ring-like swelling, but *Priesneria kellyana* has only one sensorium on antennal segment III and two sensoria on segment IV, and a glandular area on abdominal sternite IX (Pitkin 1973; Mound and Minaei 2007) which can be used for differentiating this new species.

## Supplementary Material

XML Treatment for Karnyothrips cyathomorphus

## References

[B1] Mirab-balou M Tong XL Feng JN Chen XX (2011) Thrips (Insecta: Thysanoptera) of China. Check List (Journal of Species Lists and Distribution) 7(6): 720-744.

[B2] Mound LA Marullo R (1996) The Thrips of Central and South America: An Introduction. Memoirs on Entomology, International 6: 1-488.

[B3] Mound LA Minaei K (2007) Australian thrips of the *Haplothrips* lineage (Insecta: Thysanoptera). Journal of Natural History 41(45–48): 2919-2978. doi: 10.1080/00222930701783219

[B4] Okajima S (2006) The Insects of Japan. Volume 2. The suborder Tubulifera (Thysanoptera). Touka Shobo Co. Ltd., Fukuoka, 720 pp.

[B5] Pitkin BR (1973) A revision of the Australian Haplothripini, with descriptions of three new species (Thysanoptera: Phlaeothripidae). Journal of the Australian Entomological Society 12: 315-339. doi: 10.1111/j.1440-6055.1973.tb01680.x

[B6] ThripsWiki (2013) ThripsWiki - providing information on the World’s thrips. http://thrips.info/wiki/ [Accessed 3 September 2013]

[B7] Watson JR (1922) Another camphor thrips. Florida Entomologist 6: 6-7. doi: 10.2307/3492799

